# *PAFAH1B1* governs follicular development by modulating the protein complex of CCNE1-CDK2-CDK1 to induce cell cycle arrest

**DOI:** 10.3389/fcell.2026.1919659

**Published:** 2026-09-01

**Authors:** Yingting He, Yinqi Zhou, Ruiqi Wang, Weili Liao, Nian Li, Jiaqi Li, Zewen Sun, Jianguo Zhao, Xiaolong Yuan

**Affiliations:** 1 State Key Laboratory of Swine and Poultry Breeding Industry, National Engineering Research Center for Breeding Swine Industry, Guangdong Provincial Key Lab of Agro-Animal Genomics and Molecular Breeding, College of Animal Science, South China Agricultural University, Guangzhou, China; 2 State Key Laboratory of Organ Regeneration and Reconstruction, Institute of Zoology, Chinese Academy of Sciences, Beijing, China; 3 National Center of Technology Innovation for Pigs, Chongqing, China

**Keywords:** CCNE1-CDK2-CDK1 complex, cell cycle, DNA methylation, follicular development, *PAFAH1B1*

## Abstract

**Background:**

Ovarian follicle development plays a crucial role in mammalian fertility, which is primarily regulated by granulosa cell (GC) proliferation and cell cycle. Cell cycle dysregulation collectively might drive follicular atresia through GC dysfunction. However, the underlying molecular mechanisms remain largely unexplored.

**Methods:**

The scRNA-seq and integrative analysis revealed that *PAFAH1B1* was involved in cell cycle. Functional assays, including overexpression/knockdown, flow cytometry, EdU, HE, and TUNEL, confirmed that *PAFAH1B1* regulated cell cycle and follicular development *in vitro* and *in vivo*. CoIP showed that *PAFAH1B1* bound CCNE1-CDK2-CDK1 to arrest G2/M phase. Chromatin accessibility and CRISPR/dCas9-TET1 demonstrated that DNA methylation modulated *PAFAH1B1* transcription.

**Results:**

A novel regulator of cell cycle, *PAFAH1B1*, was identified in Pig Genotype-Tissue Expression (PigGTEx). During GC proliferation, we found that *PAFAH1B1* transcription was correlated with the distribution rate of G1 phase in GCs. PAFAH1B1 protein was confirmed to specifically bind to CCNE1-CDK2-CDK1 to arrest G2/M phase. Notably, *PAFAH1B1* appeared to hinder the development of follicles. Furthermore, the demethylation significantly promoted the transcription activity and chromatin accessibility of CpG island (−7 bp to +170 bp) of *PAFAH1B1*. Taken together, *PAFAH1B1* physically interacted with the CCNE1-CDK2-CDK1 complex to arrest G2/M phase and inhibit the GCs proliferation and follicular development. Additionally, demethylation of CpG island significantly promoted the transcription of *PAFAH1B1*.

**Conclusion:**

These findings not only advance understanding of cell proliferation and cycle regulation but also identify *PAFAH1B1* as a candidate gene for further investigation in follicular development.

## 1 Introduction

Subfertility or infertility is a growing reproductive problem and affects approximately one in six couples worldwide (Farquhar et al., 2019). Normal ovarian development and granulosa cell (GC) proliferation are essential for proper functioning of the reproductive system. GCs, as the main components of follicles, play an essential role in follicular development by secreting growth factors and hormones (Fan et al., 2009). Dysfunction of GCs, characterized by impaired steroidogenesis and increased apoptosis (Wang et al., 2023; Zhang et al., 2018), ultimately causes follicular developmental defects and impaired fertility. Understanding the regulatory mechanisms of GCs and follicular development is beneficial for identifying new targets to treat subfertility or infertility.

The normal cell cycle of GCs is crucial for follicular development and maturation. Cyclin E1 (*CCNE1*) is a critical cell cycle regulator that forms a complex with cyclin-dependent kinase 2 (*CDK2*) and facilitates G1/S transition (Karst et al., 2014). CCNE1 binds to and activates CDK1, which acts as the catalytic subunit of highly conserved M-phase promoting factor complex, a key regulator essential for the G2/M phase transition (Gallo et al., 2022). Recently, increasing evidence has confirmed that DNA methylation regulates GCs function and follicular development through mediating the expression of essential genes (Zhou et al., 2021; Zhou et al., 2023). In mammals, DNA methylation regulated *RSPO2*, thereby promoting GCs proliferation and follicular development (Zhou et al., 2021). In GCs of preovulatory follicles, changes in DNA methylation patterns cause significant shifts in function (Kawai et al., 2018). However, the mechanisms by which DNA methylation regulate GC proliferation and cell cycle remain uncharacterized.

Platelet activating factor acetylhydrolase 1B regulatory subunit 1 (*PAFAH1B1*), is the regulatory subunit of PAFAH1B complex that inactivates platelet activating factor (PAF) (Tjoelker and Stafforini, 2000; Yao et al., 2015). Previous studies have shown that *PAFAH1B1* regulates the cell apoptosis (Feng et al., 2022) and cell cycle (Kardon and Vale, 2009) in various cells. It suppresses cell proliferation in cholangiocarcinoma (Yang et al., 2014) and protects osteoclast precursors from apoptosis (Ye et al., 2016). Our previous study showed that DNA methyltransferase inhibitor (5-aza-CdR) inhibits cell proliferation, promotes apoptosis in GCs, and inhibits follicular development (Zhou et al., 2023). Bioinformatic analysis identified three CpG islands (CGIs) in the *PAFAH1B1* promoter region, suggesting that its transcription may be regulated by DNA methylation. Therefore, DNA methylation may regulate the transcription of *PAFAH1B1* to mediate the cell cycle progression, thereby influencing both GC proliferation and follicular development. We further investigated the impact of *PAFAH1B1* on the cell cycle in GCs and further explored its potential mechanism in regulating cell cycle progression and follicular development. Additionally, the specific mechanism by which DNA methylation regulates the transcription of *PAFAH1B1* was elucidated. Our findings suggested that *PAFAH1B1* was a potential molecular target for improving follicular development.

## 2 Materials and methods

### 2.1 The single-cell RNA sequencing (scRNA-seq) and data analysis

The slaughtering experiment and library preparation of scRNA-seq were performed as preciously described (Chen et al., 2023). Raw data (GEO: GSE237134) were processed with CellRanger v6.0.2 (10X Genomics) using the Sscrofa11.1 pig genome (GCA_000003025.6) for alignment, filtering, and UMI counting. The R packages Seurat (v4.3.0.1) and Harmony (v0.1.1) (Pertea et al., 2015) were utilized for data filtering, normalization, dimensionality reduction, clustering, and downstream analyses. For data filtering, cells with gene counts below 5,000 and mitochondrial content below 30% were retained to exclude potentially low-quality or damaged cells. For dimensionality reduction and clustering, the top 2,000 highly variable genes were selected for principal component analysis, and the top 30 principal components were retained for subsequent analyses. Graph-based clustering algorithms were applied for cell clustering, and t-distributed stochastic neighbor embedding (t-SNE) and uniform manifold approximation and projection (UMAP) were used for visualization. Upon integration of the datasets with R package Harmony, we identified four clusters, which were subsequently annotated into four major cell types (theca cells, mural GCs, cumulus GCs, and immune cells) based on cell-type-specific marker genes. Marker genes specific to theca cells (*STAR*), mural GCs (*AREG* and *CYB5A*), cumulus GCs (*VCAN*), and immune cells (*CTSS*, *FCER1G*, *C1QB*, and *C1QC*) were utilized. Differentially expressed genes (DEGs) were identified with FindMarkers (|log_2_ (fold change) |>1 and *P* < 0.05). Enrichment analyses were subsequently performed across the identified cell types. Gene set scores for pathways including ovarian steroidogenesis, cell cycle, and apoptosis were calculated using Seurat’s AddModuleScore.

### 2.2 Integrative analysis of *PAFAH1B1* in ovarian development

Using data from Pig Genotype-Tissue Expression (PigGTEx) resource (http://piggtex.farmgtex.org) (Teng et al., 2024), we selected the top 100 genes positively correlated with *PAFAH1B1* expression. Using FindMarkers, DEGs were called at a threshold of |log_2_ (fold change) |>1 and *P* <0.05. GO enrichment analysis was performed. By integrating eQTL data, we analyzed genetic associations between these regulatory loci and six reproductive traits, including total litter birth weight and age at first farrowing. Tissue-specific *PAFAH1B1* expression was profiled across 15 organs.

### 2.3 Cell culture

COV434 cells (Cat. No. CL-0527, Procell, China), which are human granulosa tumor cells, were cultured in DMEM (Invitrogen, United States) with 10% FBS, 100 IU/mL penicillin, and 100 μg/mL streptomycin. Incubation conditions were set at 37 °C and 5% CO_2_. Plasmid transfection was achieved using Lipofectamine™ 3000 (Thermo Fisher, MA, United States) for 24 h, followed by 0.5 μM 5-Aza-CdR treatment for 24 h.

### 2.4 Follicle culture

Ovaries were purchased from a notable slaughterhouse (Guangzhou, China). Healthy 3–5 mm follicles were isolated from the ovaries. Isolated antral follicles were cultivated in a 24-well plate using serum-free DMEM/F12 medium. The culture was maintained at a temperature of 38.5 °C and 5% CO_2_. Lentiviral vectors overexpress or interfere with *PAFAH1B1* (LV-*PAFAH1B1* or sh-*PAFAH1B1*) were synthesized by Dongze Biotechnology Co., Ltd. (Guangzhou, China) at a titer of 1*10^8 TU/mL. Isolated follicles (n = 12 per group) were injected with 3 × 10^7^ TU (n = 8) lentivirus.

### 2.5 Animal experiment

A total of 32 female C57BL/6J mice (three-weeks-old) were obtained from Guangdong Medical Laboratory Animal Center (Guangzhou, China). These mice were maintained in air-conditioned rooms with adequate water and food. Subsequently, they were randomly assigned into four groups (n = 8 per group): LV-*Pafah1b1*, LV-NC, sh-*Pafah1b1*, and sh-NC. Lentiviral vectors overexpress or interfere with *Pafah1b1* (LV-*Pafah1b1* or sh-*Pafah1b1*) were synthesized by Dongze Biotechnology Co., Ltd. (Guangzhou, China) at a titer of 1*10^8 TU/mL. Each group of mice were intraperitoneally injected with 1 × 10^7^ TU lentivirus once a week for three consecutive weeks. The developmental period of the mice was recorded, marked by the opening of the vagina. From each group, three mice were randomly selected for subsequent analyses. The randomization sequence was generated using Microsoft Excel software. The animals were housed in temperature-controlled rooms with *ad libitum* access to water and food. Anesthesia was induced by intraperitoneal injection of 10% chloral hydrate at a dose of 5 mL/kg body weight.

### 2.6 Hematoxylin and eosin staining

Sections were baked at 60 °C for 20 min, deparaffinized in xylene (2 × 10 min), rehydrated in 95% and 75% ethanol (5 min each), and rinsed with distilled water. Nuclear staining used hematoxylin (10 min), followed by tap water rinse until blue. Cytoplasmic staining used eosin (3 min). Sections were dehydrated in 95% and 100% ethanol (5 min each), cleared in xylene (2 × 10 min), and mounted with mounting medium. Images were taken with a Nikon ECLIPSE Ti2 microscope. Count the number of preantral follicles (PF), antral follicles (AF), corpus luteum (CL) and the total number of follicles (TNF) per ovary. Follicles were classified morphologically.

### 2.7 TUNEL assay

42-day-mice from each treatment group were euthanized. Their ovaries were harvested for subsequent TUNEL assay performed according to the Kit instruction (Roche Applied Science, Shanghai, China). Paraffin-embedded ovaries were sectioned at 6 µm. Deparaffinized and rehydrated sections were treated with 200 μg/mL proteinase-K for 15 min at 37 °C, then washed with phosphate-buffered saline (PBS) for 20 min. Sections were finally counterstained with 4′, 6-diamidino-2-phenylindole. Images were captured using a laser scanning confocal microscope (Zeiss, Oberkochen, Germany). TUNEL positive follicles were counted in sections taken through the largest diameter of each ovary.

### 2.8 Western Blot (WB)

Cells were washed, collected, and lysed on ice for 30 min in M-per lysis buffer (Thermo Fisher, MA, United States) containing protease and phosphatase inhibitors (Protein Halts, Thermo Fisher, MA, United States). Equal amounts of protein were separated and electro transferred to polyvinylidene difluoride membrane (Millipore, Darmstadt, Germany). Membranes were blocked and incubated at 4 °C with primary antibodies overnight. Membranes were incubated with secondary antibodies after washing. Bands were detected using chemiluminescent substrate (Thermo Fisher, MA, United States) and quantified by densitometry with ImageJ. The primary antibodies: anti-PAFAH1B1 (sc-374586, Santa cruz, United States), anti-PCNA (10205-2-AP, Proteintech, China), anti-GSK3B (82061-1-RR, Proteintech), anti-CASP3 (66470-2-Ig, Proteintech), anti-CASP9 (10380-1-AP, Proteintech), anti-P53 (10442-1-AP, Proteintech), anti-CDK1 (19532-1-AP, Proteintech), anti-CDK2 (10122-1-AP, Proteintech), anti-CDK4 (11026-1-AP, Proteintech), anti-CCND1 (60186-1-Ig, Proteintech), anti-CCNB1 (55004-1-AP, Proteintech), anti-CCNB2(21644-1-AP, Proteintech), anti-CCNE1 (11554-1-AP, Proteintech), anti-CCNE2 (11935-1-AP, Proteintech), anti-Phospho-CDK1 (YP0054, Immunoway, United States), anti-Phospho-CDK2 (YP0592, Immunoway), anti-pH3S10 (YT5822, Immunoway), anti-α-Tubulin (11224-1-AP, Proteintech), and anti-GAPDH (ab8245, Abcam, United Kingdom). Secondary antibodies: anti-Rabbit IgG (L3012, Signalway Antibody, China) or anti-mouse IgG (L3012, Signalway Antibody).

### 2.9 Quantitative real-time PCR (qRT-PCR)

Total RNA was isolated from mouse ovarian and GCs using TRIZOL (Invitrogen, United States). RNA concentration was determined by measuring the OD260/OD280 ratio using spectrophotometry. The integrity of RNA was evaluated through electrophoresis on a 1% formaldehyde denaturing agarose gel. To generate cDNA samples, the Hifair® AdvanceFast 1st Strand cDNA Synthesis Kit (Yisheng Bio-Technology, Shanghai, China) was utilized. The Hieff® qPCR SYBR Green Master Mix (Yisheng Bio-Technology, China) was utilized. Real-time PCR detection was performed by the CFX96 Touch Real-Time PCR system (Bio-Rad, United States). To ensure reliable normalization, GAPDH was used as an endogenous control. The relative levels of mRNAs were determined by 2^−ΔΔCT^ method. The primer used for qRT-PCR in the human (Table 1), pig (Table 2), and mouse (Table 3) were shown below.

**TABLE 1:** Primers used for qPCR in humans.

Primer name	Sequence (5′-3′)
PAFAH1B1-F	AGC​GAT​GCA​TGA​AGA​CCC​TC
PAFAH1B1-R	TGA​TCT​ACG​CTG​CCA​GTG​AC
GAPDH-F	TGT​TCG​TCA​TGG​GTG​TGA​AC
GAPDH-R	ATG​GCA​TGG​ACT​GTG​GTC​AT
CDK1-F	CTG​GGG​TCA​GCT​CGT​TAC​TC
CDK1-R	TCC​ACT​TCT​GGC​CAC​ACT​TC
CDK2-F	ATG​GAT​GCC​TCT​GCT​CTC​ACT​G
CDK2-R	CCC​GAT​GAG​AAT​GGC​AGA​AAG​C
CDK4-F	CCA​TCA​GCA​CAG​TTC​GTG​AGG​T
CDK4-R	TCA​GTT​CGG​GAT​GTG​GCA​CAG​A
CCND1-F	TCT​ACA​CCG​ACA​ACT​CCA​TCC​G
CCND1-R	TCT​GGC​ATT​TTG​GAG​AGG​AAG​TG
CCNB1-F	TGC​AGC​ACC​TGG​CTA​AGA​AT
CCNB1-R	TAG​CAT​GCT​TCG​ATG​TGG​CA
CCNB2-F	CAA​CCA​GAG​CAG​CAC​AAG​TAG​C
CCNB2-R	GGA​GCC​AAC​TTT​TCC​ATC​TGT​AC
CCNE1-F	TGC​AGC​ACC​TGG​CTA​AGA​AT
CCNE1-R	TAG​CAT​GCT​TCG​ATG​TGG​CA
CCNE2-F	CTT​ACG​TCA​CTG​ATG​GTG​CTT​GC
CCNE2-R	CTT​GGA​GAA​AGA​GAT​TTA​GCC​AGG

**TABLE 2:** Primers used for qPCR in pigs.

Primer name	Sequence (5′-3′)
PAFAH1B1-F	CTG​ATG​ACA​AGA​CCC​TGC​GT
PAFAH1B1-R	GGC​ACT​CCC​ACA​CCT​TTA​CT
GAPDH-F	GGA​CTC​ATG​ACC​ACG​GTC​CAT
GAPDH-R	TCA​GAT​CCA​CAA​CCG​ACA​CGT
CDK1-F	AAG​TGT​GGC​CAG​AAG​TGG​AG
CDK1-R	CCA​GAA​ATT​CGC​TTG​GCA​GG
CDK2-F	TTT​GCT​GAG​ATG​GTG​ACT​CG
CDK2-R	TGT​TAG​GGT​CGT​AGT​GCA​GC
CCNE1-F	GGA​TTT​TTA​AAT​GTC​CCG​CTC​T
CCNE1-R	GGT​TCC​TTC​GCA​TCC​CTT​TC
CCNE1-F	GGA​TTT​TTA​AAT​GTC​CCG​CTC​T
CCNE1-R	GGT​TCC​TTC​GCA​TCC​CTT​TC

**TABLE 3:** Primers used for qPCR in mice.

Primer name	Sequence (5′-3′)
PAFAH1B1-F	GCG​AAC​TCT​CAA​GGG​CCA​TA
PAFAH1B1-R	CAT​TGT​GAT​CGT​GAC​CGT​GC
GAPDH-F	GGT​CCC​AGC​TTA​GGT​TCA​TCA
GAPDH-R	CCA​ATA​CGG​CCA​AAT​CCG​TT
CDK1-F	CAT​GGA​CCT​CAA​GAA​GTA​CCT​GG
CDK1-R	CAA​GTC​TCT​GTG​AAG​AAC​TCG​CC
CDK2-F	TCA​TGG​ATG​CCT​CTG​CTC​TCA​C
CDK2-R	TGA​AGG​ACA​CGG​TGA​GAA​TGG​C
CCNE1-F	AAG​CCC​TCT​GAC​CAT​TGT​GTC​C
CCNE1-R	CTA​AGC​AGC​CAA​CAT​CCA​GGA​C
CCNE1-F	GCG​AAC​TCT​CAA​GGG​CCA​TA
CCNE1-R	CAT​TGT​GAT​CGT​GAC​CGT​GC

### 2.10 EdU assay

Cell-Light^TM^ Edu Apollo (RiboBio, China) was used to detect the GC proliferation. GCs were transfected with plasmid for 24 h, then incubated with 50 μM EDU regent for 2 h. GCs fixed, treated with 0.5% Triton X-100, and then stained with Apollo and Hoechst for 30 min in darkness respectively. Data analysis was performed under an inverted fluorescence microscope to count the number of germinal centers in three randomly selected fields. The proliferation rate of GCs was calculated using the formula: proliferating cells labeled with EdU divided by the total number of cells labeled with Hoechst.

### 2.11 Flow cytometry assay

Cellular apoptosis of GCs was assessed using an Annexin V-FITC/PI Kit (BD Biosciences, Franklin Lakes, United States). COV434 cells were collected and pelleted 24 h post-transfection or drug treatment. After washing, the cells resuspended in 1× Annexin V buffer. The cells were subsequently stained with Annexin V-FITC and PI in the dark. Quadrants were defined as Q1 (necrotic cells), Q2 (late apoptotic cells), Q3 (early apoptotic cells), and Q4 (viable cells). The percentage of apoptosis cells was calculated as the sum of late-stage (Q2 levels) and early-stage (Q3 levels) apoptotic cells.

### 2.12 Cell cycle assay

COV434 cells were cultured in serum-free DMEM medium for 24 h to induce G0/G1 cell cycle arrest. This serum starvation protocol has been widely employed for cell cycle synchronization in various cell types (Du et al., 2016; Han et al., 2020; Takeuchi et al., 2014). Following synchronization, the cells were re-stimulated with complete medium with 10% FBS and collected at 0, 4, 8, 12, 16, 20, and 24 h. To explore the influence of cell culture density on the cell cycle progression, COV434 cells were synchronized using the same serum starvation protocol. Subsequently, GCs were collected at different densities, including 50%, 80%, 100%, and over confluence (OC). The cells used to assess the impact of *PAFAH1B1* on cell cycles were initially seeded in a 6-well plate and cultured until reaching 50%–80% confluency. To detach the cells, trypsin solution without EDTA was used, and then the cells were washed with 2 mL of PBS solution. For fixation, the cells were treated with 70% ethanol and kept at 4 °C for at least 4 h. Then washed the cells twice. Cells were gently resuspended in 400 µL of ethidium bromide solution and 100 µL of RNase was added. The mixture was then incubated at 4 °C in the dark for 30 min.

### 2.13 CoIP assay

CoIP kit (Millipore, United States) was used to perform the CoIP assay. Collected cells were subjected to immunoprecipitation at 4 °C overnight with PAFAH1B1. Additionally, antibodies against CCND1, CCNB1, CCNB2, CCNE1, and CCNE2 were used for negative verification. IgG was used as control. The accumulated proteins were detected by WB.

### 2.14 Dual-luciferase reporter assay

The NCBI website was used for predicting the promoter region of *PAFAH1B1*. Within the 2201 bp (−2000 bp to +200 bp) sequence of *PAFAH1B1* promoter, three high CGIs were observed on −880 bp to −592 bp, −506 bp to −296 bp, and −7 bp to +170 bp. The 2201 bp sequence was divided into five deletion fragments, named CGI1 (−880 bp to −592 bp), CGI2 (−506 bp to −296 bp), CGI3 (−7 bp to +170 bp), NCGI1 (−1135 bp to −930 bp), and NCGI2 (−318 bp to −51 bp). Fragments were cloned into pGL3 vector to obtain pGL3-CGI1, pGL3-CGI2, pGL3-CGI3, pGL3-NCGI1, and pGL3-NCGI2. The specific primes were listed in Table 4. Transfected si-DNMT3A to COV434, we detected the luciferase activities by Dual-luciferase Reporter Assay Kit (Promega, WI, United States).

**TABLE 4:** Primers used for vector construction.

Primer name	Sequence (5′-3′)
OE-PAFAH1B1-F	CGG**GGT​ACC**GCG​CTC​CGG​TGG​AAT​G
OE-PAFAH1B1-R	CTA**GCT​AGC**AGG​AGG​GGC​CAA​ATG​AGA​CT
pGL3-CGI1-F	CG**ACG​CGT**TTG​AAT​TAT​TTA​GAA​TTT​TAA​ATT​T
pGL3-CGI1-R	CC**CTC​GAG**TAC​AAT​CTC​CAC​TCA​CTA​CAA​ACT​CC
pGL3-CGI2-F	CG**ACG​CGT**TGG​TAA​GTG​TTT​GTA​TTT​TTA​GTT​ATT
pGL3-CGI2-R	CC**CTC​GAG**TCT​TAA​AAA​TTT​ATA​ATT​AAT​TTA​TTC​CAC
pGL3-CGI3-F	CG**ACG​CGT**AAT​TAT​TTC​GGA​GGT​CGG​AGG
pGL3-CGI3-R	CC**CTC​GAG**CTA​AAC​TCT​AAC​TCT​ACT​CAC​CTC​G
pGL3-NCGI1-F	CG**ACG​CGT**GAG​GTA​TGG​TAA​TTT​GTT​TTA​GGT
pGL3-NCGI1-R	CC**CTC​GAG**CAT​TTA​TAT​TAT​CCA​AAA​AAA​CTT​TTT
pGL3-NCGI2-F	CG**ACG​CGT**GGA​ATA​AAT​TAA​TTA​TAA​ATT​TTT​AAG​A
pGL3-NCGI2-R	CC**CTC​GAG**TTA​AAA​TTA​AAA​AAC​AAA​TAA​AAT​ACT​ATC

Sequences bold represent the enzyme cutting sites.

### 2.15 Chromatin accessibility assay

After transfected si-DNMT3A into COV434 for 24 h, EpiQuik™ Chromatin Accessibility Assay Kit (Epigentek, United States) were utilized to assess the chromatin accessibility of the promoter of *PAFAH1B1* (−2000 bp to +200 bp). We collected the processed GCs, performed chromatin digestion, isolated DNA, conducted q-PCR, and then analyzed q-PCR data. The specific primes for PCR were listed in Table 5.

**TABLE 5:** Primers used for chromatin accessibility and BSP.

Primer name	Sequence (5′-3′)
CGI1-F	TTG​AAT​TAT​TTA​GAA​TTT​TAA​ATT​T
CGI1-R	TAC​AAT​CTC​CAC​TCA​CTA​CAA​ACT​CC
CGI2-F	TGG​TAA​GTG​TTT​GTA​TTT​TTA​GTT​ATT
CGI2-R	TCT​TAA​AAA​TTT​ATA​ATT​AAT​TTA​TTC​CAC
CGI3-F	AAT​TAT​TTC​GGA​GGT​CGG​AGG
CGI3-R	CTA​AAC​TCT​AAC​TCT​ACT​CAC​CTC​G
NCGI1-F	GAG​GTA​TGG​TAA​TTT​GTT​TTA​GGT
NCGI1-R	CAT​TTA​TAT​TAT​CCA​AAA​AAA​CTT​TTT
NCGI2-F	GGA​ATA​AAT​TAA​TTA​TAA​ATT​TTT​AAG​A
NCGI2-R	TTA​AAA​TTA​AAA​AAC​AAA​TAA​AAT​ACT​ATC
CGI3-BSP-F	AAT​TAT​TTC​GGA​GGT​CGG​AGG​CAG​A
CGI3-BSP-R	CTA​AAC​TCT​AAC​TCT​ACT​CAC​CTC​G

### 2.16 Bisulfite sequencing PCR (BSP)

Genome DNA was extracted by a Tissue DNA Kit (Omega Bio-tek, United States). The purified DNA was then bisulfite-converted with an EZ DNA Methylation-Gold ^TM^ Kit (ZYMO, United States) to achieve CT conversion and amplified with BSP primer. QUMA was used to compare the sequencing data with the original sequence, from which dot plots were generated. For methylation rate analysis, a minimum of 10 clones per sample were required as preciously described (Chen et al., 2024; Zhou et al., 2023). To demethylate the CGI3 region of the *PAFAH1B1* promoter (−7 bp to +170 bp), the dCas9-TET1 vector and two synthetic gRNAs (Dongze Biotechnology, China) were employed. The target sequence of gRNA1 was GCC​AAC​GGG​ACG​CCG​CGG​TCG​G, while the target sequence of gRNA2 was GCG​CTC​ATG​CGG​CCG​ACC​GCG​G.

### 2.17 Statistical analysis

All data are expressed as the mean ± SEM of at least three independent experiments and were performed in triplicate. Student’s t-test (two-sided) was used to measure differences. *p* < 0.05 and *p* < 0.01 were considered statistically significant. *, *p* < 0.05. **, *p* < 0.01.

## 3 Results

### 3.1 The cell cycle dysregulation of GCs drove follicular atresia

**FIGURE 1:**
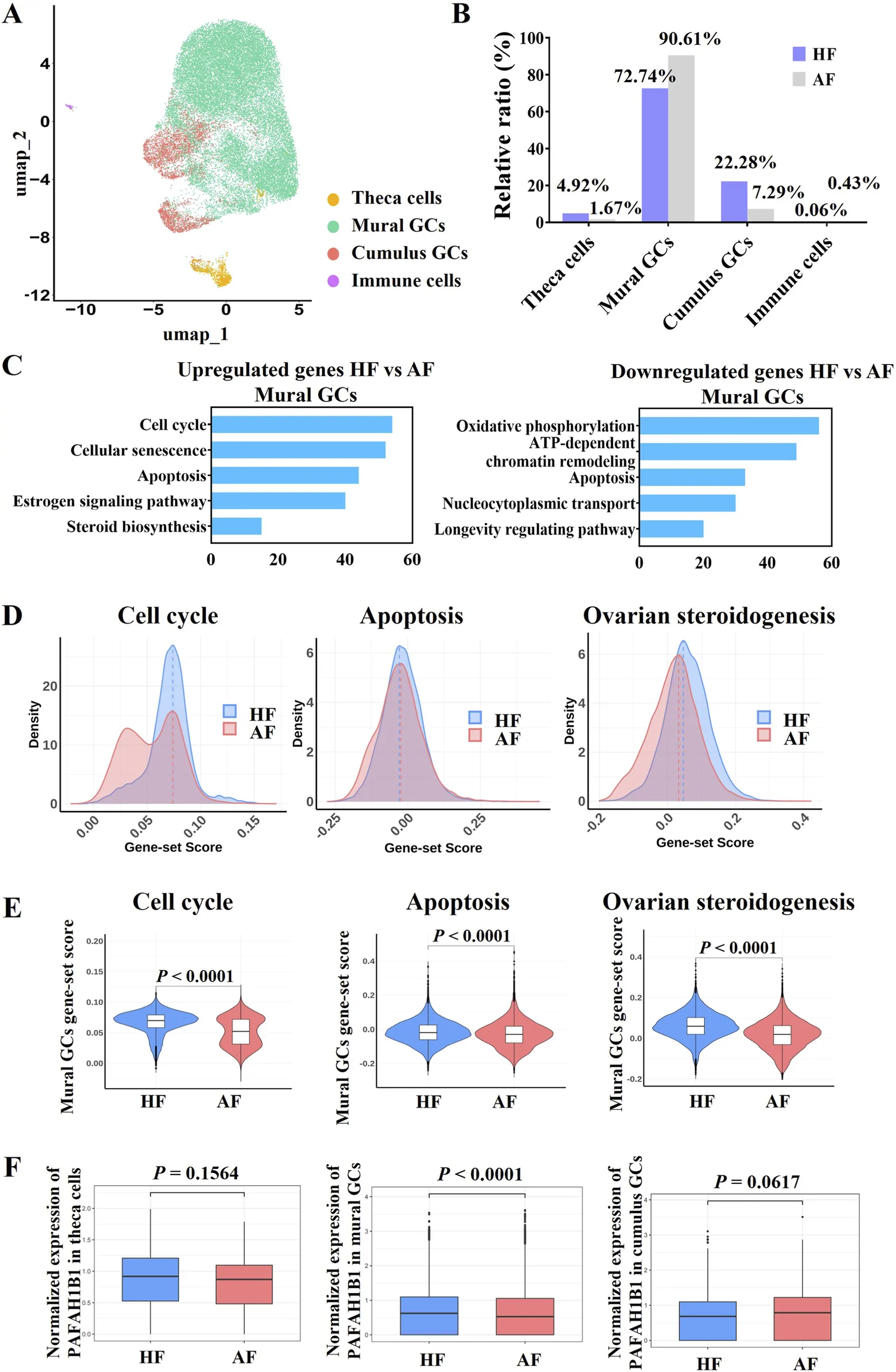
The single-cell transcriptomes between healthy and atretic follicles. **(A)** UMAP plot depicting four ovarian cell types in healthy vs. atretic porcine follicles. **(B)** Proportions of the four cell types in healthy follicles (HF) and atretic follicles (AF) determined by scRNA-seq. **(C)** Representative pathways enriched in DEGs of mural GCs in HF and AF and identified through functional enrichment analysis. **(D)** Ridge plot illustrating shifts in variant cell cycle, apoptosis, and ovarian steroidogenesis gene-set scores in mural GCs of HF and AF. **(E)** Violin Diagram in cell cycle, apoptosis, and ovarian steroidogenesis in mural GCs of HF and AF. **(F)** Normalized expression of *PAFAH1B1* in theca cells, mural GCs, and cumulus GCs of HF and AF.

Previously, we profiled the single-cell transcriptomes of GCs from healthy and atretic follicles in sows (Chen et al., 2023). In this study, we further refined the cellular subpopulations into theca cells, mural GCs, cumulus GCs, and immune cells (Figure 1A). We found that mural GCs showed the highest abundance among all cell types and exhibited the greatest proportional change between healthy and atretic follicles (Figure 1B). DEGs of mural GCs were enriched in cell cycle, apoptosis, estrogen signaling, and oxidative phosphorylation pathways (Figure 1C). In atretic follicles, the expressions of cell cycle, apoptosis, and ovarian steroidogenesis-related genes were notably reduced in mural GCs (Figures 1D,E). We also found that *PAFAH1B1* expression was significantly reduced in mural GCs from atretic follicles compared with healthy follicles, while no significant changes were observed in theca cells or cumulus GCs (Figure 1F). This cell-type-specific downregulation during follicular atresia suggested *PAFAH1B1* may influence follicular degeneration.

### 3.2 *PAFAH1B1* was involved in regulating the cell cycle of GCs

**FIGURE 2:**
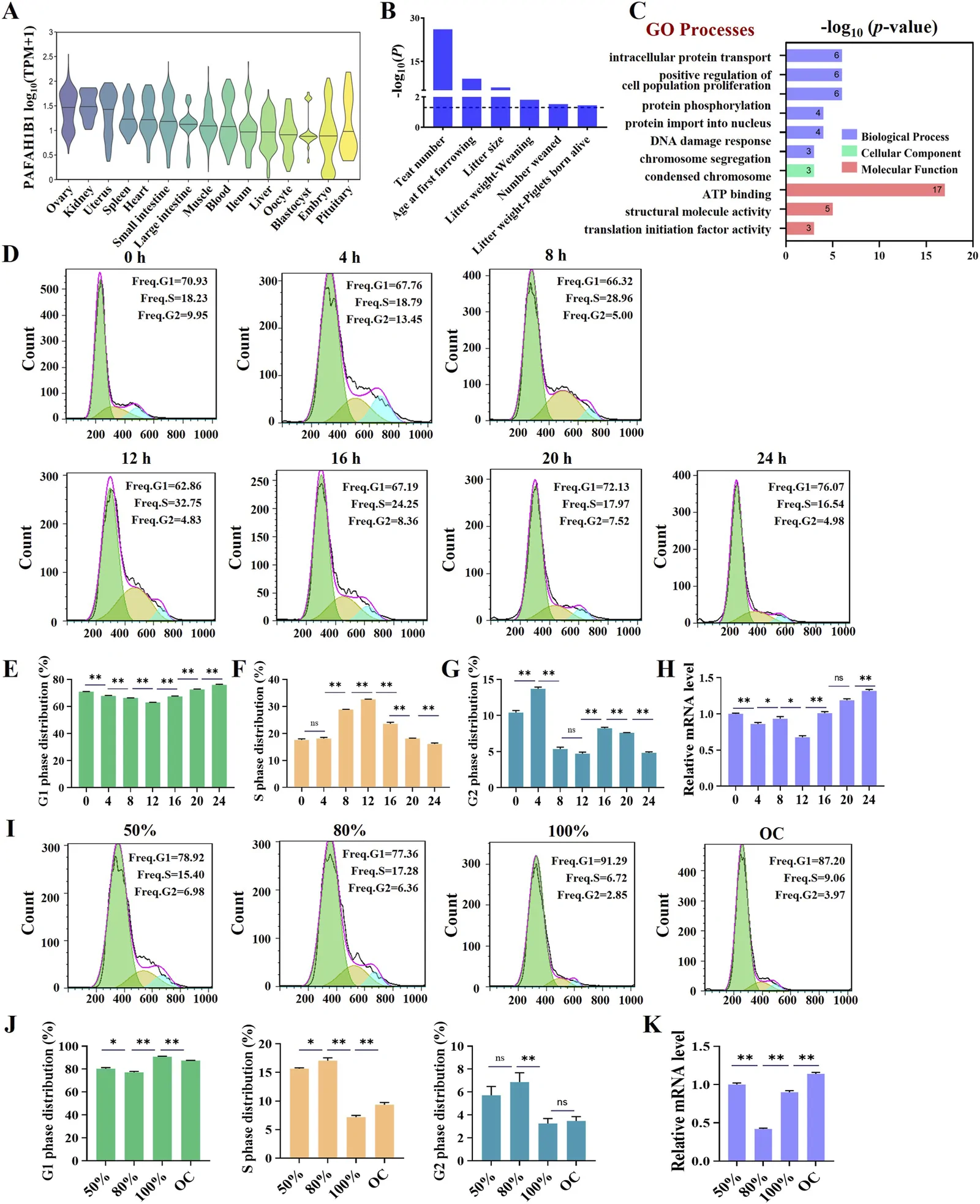
*PAFAH1B1* is involved in regulating the cell cycle. **(A)** Tissue expression profile of *PAFAH1B1* in porcine tissues (RNA-seq data from PigGTEx project. **(B)** Significant traits associations for *PAFAH1B1* in eQTLs. **(C)** GO enrichment analysis of genes positively correlated with *PAFAH1B1* expression in pig. **(D)** Flow cytometric analysis of cell cycle in GCs at different culture times (0, 4, 8, 12, 16, 20, 24 h). **(E–G)** Percentages of GCs in G1 **(E)**, S **(F)**, and G2 **(G)** phase at different culture times. **(H)** Relative mRNA level of *PAFAH1B1* in GCs at different culture times. **(I)** Flow cytometric analysis of cell cycle in GCs seeded at different densities (50%, 80%, 100%, OC). **(J)** Percentages of GCs in G1, S, and G2 phase seeded at different densities. **(K)** Relative mRNA level of *PAFAH1B1* in GCs at different seeding densities. Data are shown as mean ± SEM from three independent biological replicates. *, *p* < 0.05. **, *p* < 0.01.

Previous studies have shown that *PAFAH1B1* regulates the cell apoptosis and cell cycle (Kardon and Vale, 2009) in various cell types, including basal radial glial cells (Wimmer et al., 2026), neural stem cells (Majmudar and Keri, 2025), and HeLa cells (Mitevska et al., 2023). However, its roles in GCs have not been explored. Based on PigGTEx (Teng et al., 2024), we found the expressions of *PAFAH1B1* were the highest in ovaries among 15 tissues (Figure 2A). PheWAS demonstrated that *PAFAH1B1* expression was significantly associated with reproduction traits of pigs, e.g., teat number, age at first farrowing, litter size, litter weight of weaning, and litter weight of piglets born alive (Figure 2B). Furthermore, the top 100 genes that were most significantly positively correlated with *PAFAH1B1* expression were notably enriched in cell population proliferation and DNA damage response pathways (Figure 2C). To further investigate the molecular mechanism underlying *PAFAH1B1*-mediated regulation of cell cycle distribution in GCs, we measured the mRNA levels of *PAFAH1B1* at 0, 4, 8, 12, 16, 20, and 24 h. We found a marked decrease in the distribution rate of G1 phase cells from 0–12 h, followed by a significant increase in the distribution rate of G1 phase cells from 12–24 h (Figures 2D,E). There was a significant increase in the S phase distribution rate from 4–12 h, followed by a marked decrease from 12–24 h (Figures 2D,F). The distribution rate of G2 phase cells significantly increased both 0–4 h and 12–16 h, while decreased from 4–12 and 16–24 h (Figures 2D,G). The mRNA level of *PAFAH1B1* significantly decreased from 0–12 h and increased in 12–24 h (Figure 2H). Additionally, GCs were collected at different growth densities: 50%, 80%, 100%, and over confluence (OC). When the growth density of GCs was from 80% to 100%, the distribution rate of G1 phase significantly increased, while the distribution rate of S and G2 phase markedly decreased. When the growth density of GCs ranged from 100% to OC, the distribution rate of G1 phase markedly decreased, while the distribution rate of S phase significantly increased (Figures 2I,J). Compared to cells at a density of 50%, *PAFAH1B1* transcription was significantly lower in cells at a density of 80% and 100%. Conversely, compared to cells at a density of 100%, *PAFAH1B1* transcription was significantly higher in cells at a density of OC (Figure 2K). Thus, *PAFAH1B1* might be involved in the regulation of specific processes of the cell cycle.

### 3.3 *PAFAH1B1* inhibited proliferation and promoted the apoptosis of GCs

**FIGURE 3:**
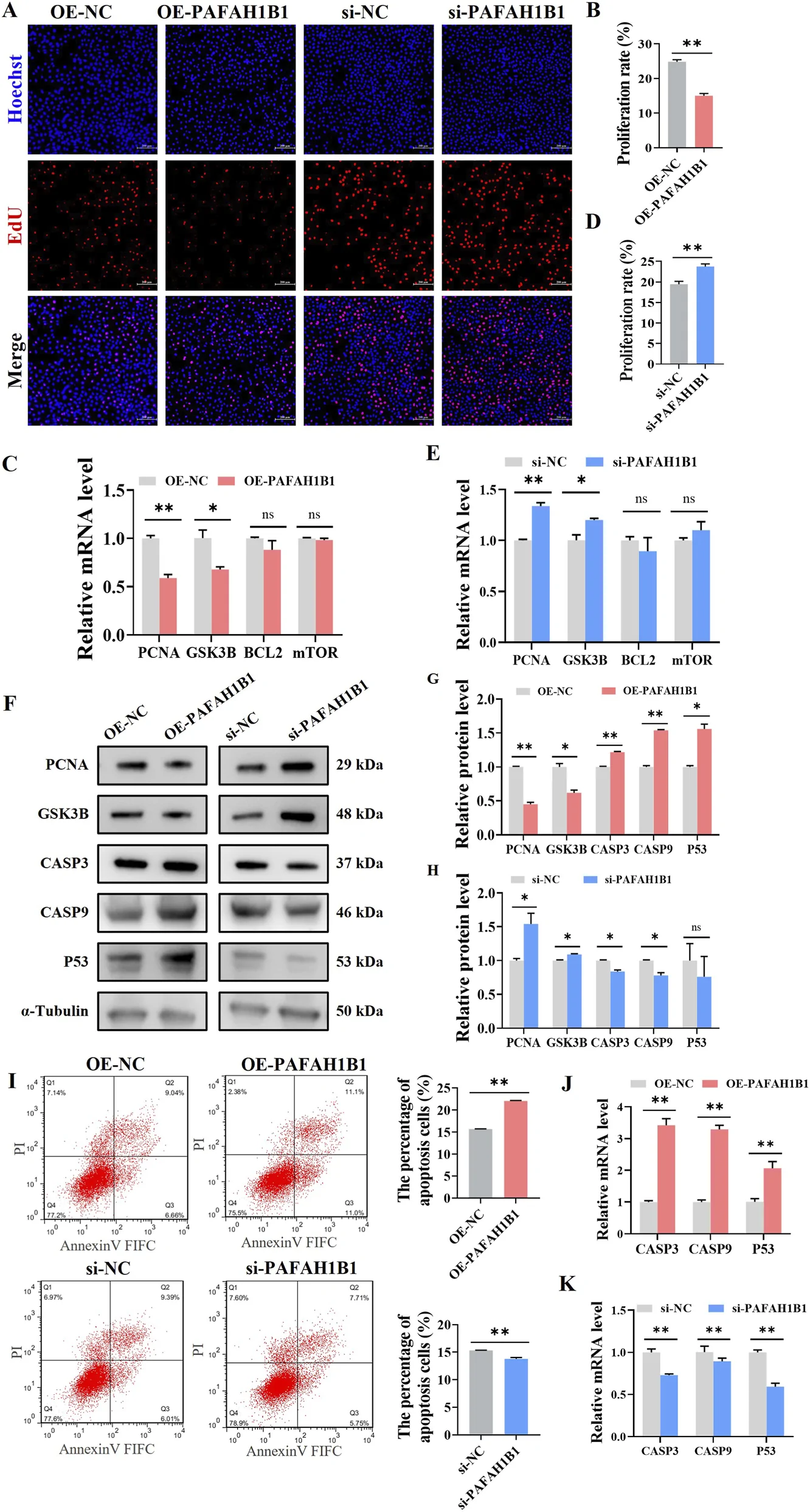
*PAFAH1B1* suppressed proliferation and promoted the apoptosis of GCs. **(A)** EdU staining (red) and DAPI nuclear staining (blue) in GCs transfected with OE-*PAFAH1B1* and si-PAFAH1B1. Scale bar, 200 μm. **(B)** Proliferation rate of GCs transfected with OE-*PAFAH1B1*. **(C)** Relative mRNA levels of *PCNA*, *GSK3B*, *BCL2* and *mTOR* in OE-*PAFAH1B1* transfected GCs. **(D)** Proliferation rate of GCs transfected with si-*PAFAH1B1*. **(E)** Relative mRNA levels of *PCNA*, *GSK3B*, *BCL2* and *mTOR* in si-*PAFAH1B1* transfected GCs. **(F)** Protein levels of PCNA, GSK3B, CASP3, CASP9, and P53 in OE-*PAFAH1B1* and si-PAFAH1B1 transfected GCs. **(G)** Relative protein levels of PCNA, GSK3B, CASP3, CASP9 and P53 in OE-*PAFAH1B1* transfected GCs. **(H)** Relative protein levels of PCNA, GSK3B, CASP3, CASP9 and P53 in si-*PAFAH1B1* transfected GCs. **(I)** Flow cytometric analysis of apoptosis in GCs transfected with OE-*PAFAH1B1* and si-PAFAH1B1. The percentage of apoptotic cells was calculated as the sum of Q2 (late apoptotic) and Q3 (early apoptotic). **(J)** Relative mRNA levels of *CASP3*, *CASP9* and *P53* in OE-*PAFAH1B1* transfected GCs. **(K)** Relative mRNA levels of *CASP3*, *CASP9* and *P53* in si-*PAFAH1B1* transfected GCs. Data are shown as mean ± SEM from three independent biological replicates. *, *p* < 0.05. **, *p* < 0.01.

To explore the potential role of *PAFAH1B1* in cell proliferation and apoptosis, GCs were treated with overexpression vectors (OE-*PAFAH1B1*) and small interfering RNAs (si-*PAFAH1B1*) targeting *PAFAH1B1*. OE-*PAFAH1B1* significantly reduced GC proliferation (Figures 3A,B) and concurrently decreased the mRNA (Figure 3C) and protein (Figures 3F,G) levels of *PCNA* and *GSK3B*. si-*PAFAH1B1* significantly increased the GC proliferation (Figures 3A,D), along with the upregulation of *PCNA* and *GSK3B* in mRNA (Figure 3E) and protein (Figures 3F,H). Besides, OE-*PAFAH1B1* significantly promoted the apoptosis (Figure 3I), accompanied by increased the expressions of *CASP3*, *CASP9* and *P53* in mRNA (Figure 3J) and protein (Figures 3F,G). si-*PAFAH1B1* significantly inhibited the apoptosis (Figure 3I), accompanied by significant downregulation of *CASP3*, *CASP9*, and *P53* at both the mRNA (Figure 3K) and protein (Figures 3F,H) levels. These results demonstrated that *PAFAH1B1* inhibited proliferation and promoted the apoptosis of GCs.

### 3.4 *PAFAH1B1* arrested GCs in the G2/M phase

**FIGURE 4:**
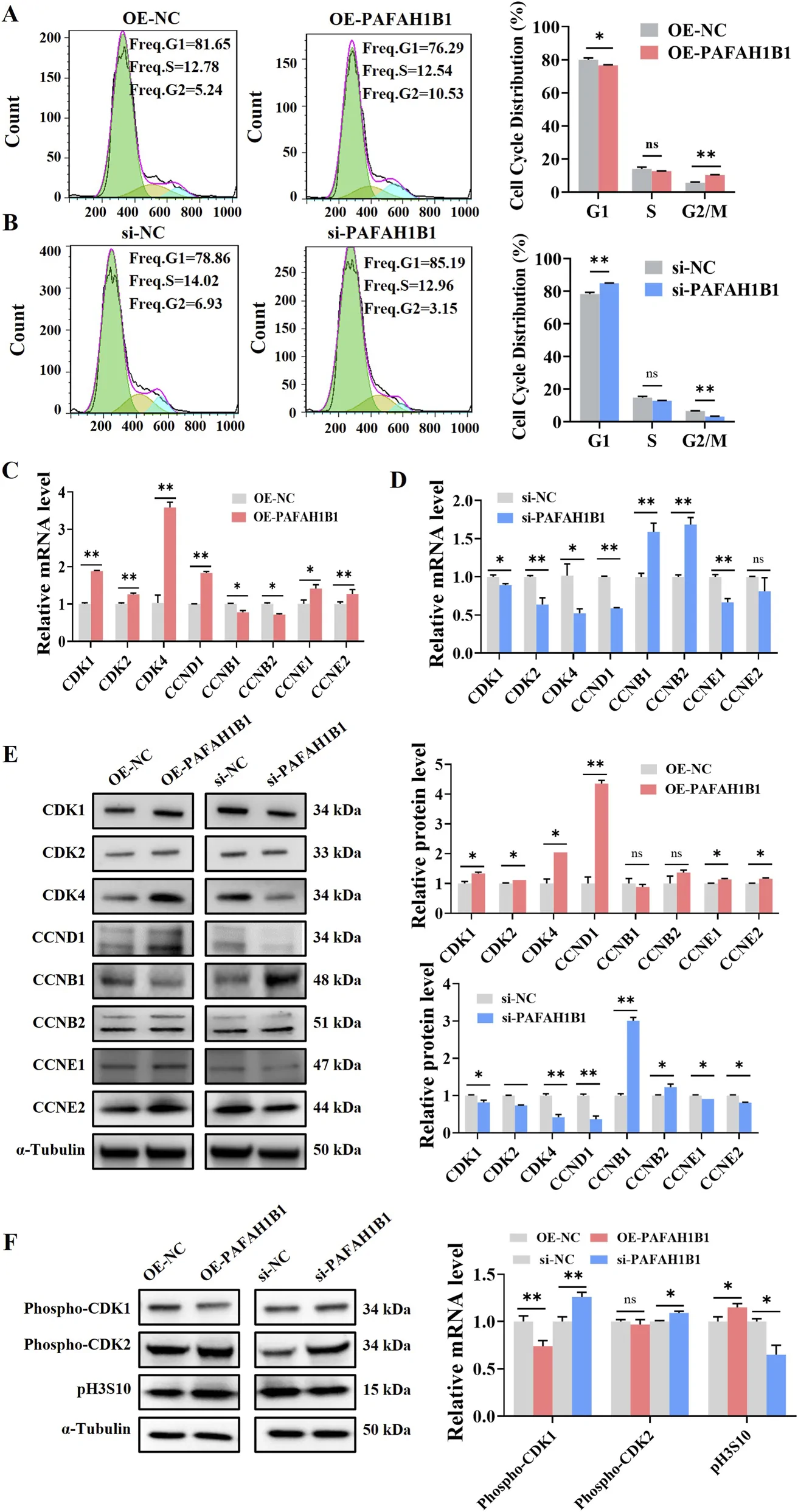
*PAFAH1B1* arrested the cell cycle at the G2/M phase in GCs. **(A)** Flow cytometric analysis of cell cycle in GCs transfected with OE-*PAFAH1B1*. **(B)** Flow cytometric analysis of cell cycle in GCs transfected with si-*PAFAH1B1.* **(C)** Relative mRNA levels of *CDK1*, *CDK2*, *CDK4*, *CCND1*, *CCNB1*, *CCNB2*, *CCNE1*, and *CCNE2* transfected with OE-*PAFAH1B1*. **(D)** Relative mRNA levels of *CDK1*, *CDK2*, *CDK4*, *CCND1*, *CCNB1*, *CCNB2*, *CCNE1*, and *CCNE2* transfected with si-*PAFAH1B1*. **(E)** Protein levels of CDK1, CDK2, CDK4, CCND1, CCNB1, CCNB2, CCNE1, and CCNE2 in OE-*PAFAH1B1* and si-PAFAH1B1 transfected GCs. **(F)** Protein levels of Phospho-CDK1, Phospho-CDK2, and pH3S10 in OE-*PAFAH1B1* and si-PAFAH1B1 transfected GCs. Data are shown as mean ± SEM from three independent biological replicates. *, *p* < 0.05. **, *p* < 0.01.

Overexpression of *PAFAH1B1* significantly decreased the distribution of GCs in G1 phase and elevated the distribution in G2/M phase relative to the control (Figure 4A), while si-*PAFAH1B1* significantly increased the distribution of GCs in G1 phase and reduced the distribution of GCs in G2/M phase (Figure 4B). In addition, OE-*PAFAH1B1* markedly increased the mRNA levels of pro-G1/S transition gene (*CDK2*, *CDK4*, *CCND1*, *CCNE1*, and *CCNE2*) and pro-G2/M phase gene (*CDK1*), while decreased the anti-G2/M phase genes *CCNB1* and *CCNB2* (Figure 4C). si-*PAFAH1B1* decreased the mRNA levels of *CDK1*, *CDK4*, *CCND1*, and *CCNE1*, but increased those of *CDK2*, *CCNB1*, and *CCNB2* (Figure 4D). OE-*PAFAH1B1* markedly promoted the protein levels of CDK1, CDK2, CDK4, CCND1, CCNE1, CCNE2 (Figure 4E), and phosphorylated histone H3 at Serine 10 (pH3S10) (Figure 4F), while significantly inhibited the protein level of Phospho-CDK1 (Figure 4F). si-*PAFAH1B1* markedly reduced the protein levels of CDK1, CDK2, CDK4, CCND1, CCNE1, CCNE2 (Figure 4E), and pH3S10 (Figure 4F) and elevated the protein level of CCNB2 (Figure 4E), Phospho-CDK1, and Phospho-CDK2 (Figure 4F). These findings suggested that *PAFAH1B1* arrested the cell cycle at the G2/M phase.

### 3.5 PAFAH1B1 bound to CCNE1-CDK2-CDK1 protein complex to arrest the cell cycle at the G2/M phase

**FIGURE 5:**
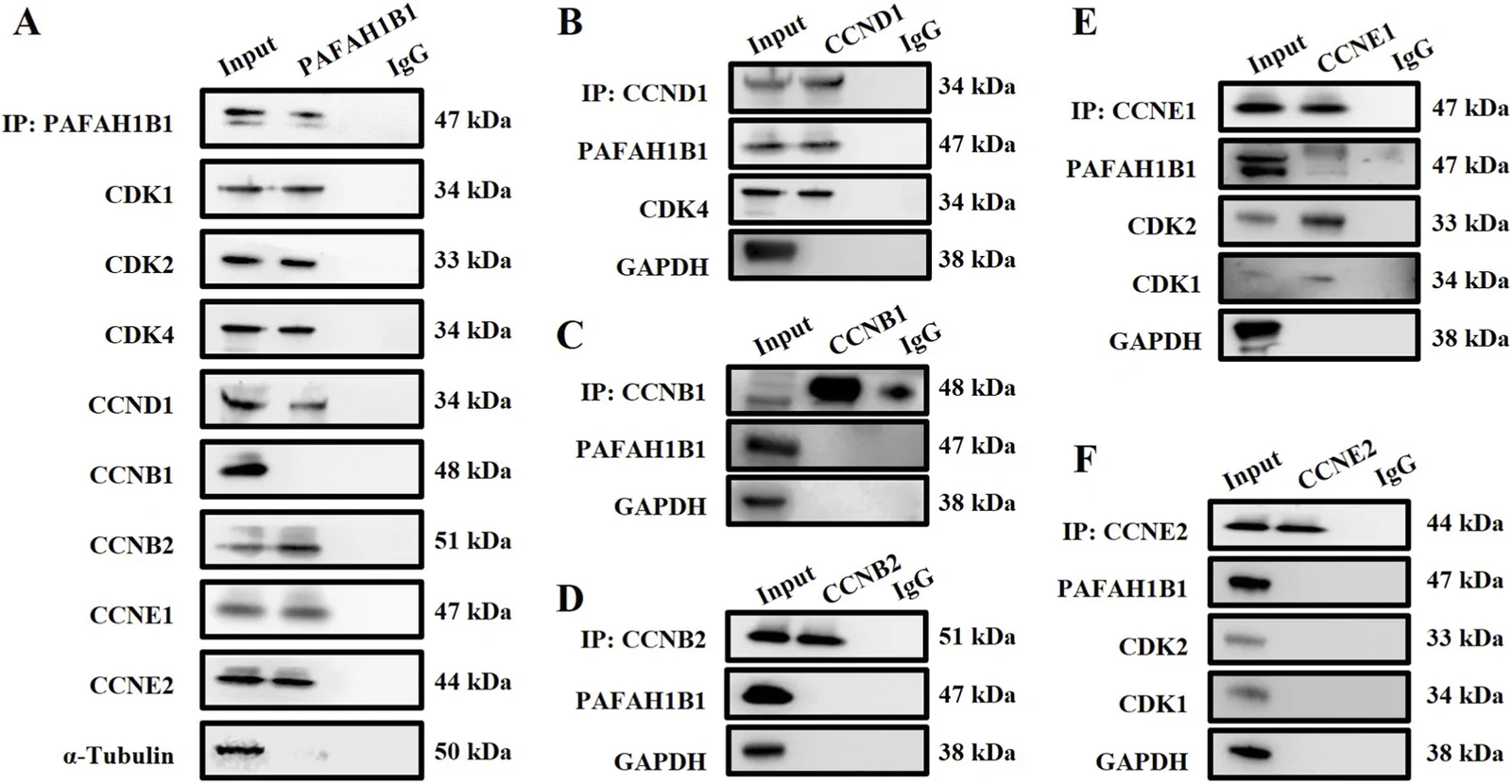
PAFAH1B1 bound to CCNE1-CDK2-CDK1 complex and arrest cell cycle at G2/M. **(A)** The binding of PAFAH1B1 and CDK1, CDK2, CDK4, CCND1, CCNB1, CCNB2, CCNE1, and CCNE2 protein detected by CoIP. **(B)** The binding of CCND1 and PAFAH1B1/CDK4 protein detected by CoIP. **(C)** The binding between CCNB1 and PAFAH1B1 protein detected by CoIP. **(D)** The binding between CCNB2 and PAFAH1B1 protein detected by CoIP. **(E)** The binding of CCNE1 and PAFAH1B1, CDK2, or CDK1 protein detected by CoIP. **(F)** The binding of CCNE2 and PAFAH1B1, CDK2, or CDK1 protein detected by CoIP.

To elucidate the potential mechanisms by which *PAFAH1B1* regulates the cell cycle, CoIP was used to detect the binding between *PAFAH1B1* and key cycle-related genes. We found that PAFAH1B1 bound to CDK1, CDK2, CDK4, CCND1, CCNB2, CCNE1, and CCNE2 proteins (Figure 5A). Reverse validation indicated that CDK4 was enriched in anti-CCND1 IP, but PAFAH1B1 could not be enriched (Figure 5B). PAFAH1B1 also could not be enriched by anti-CCNB1 (Figure 5C) and anti-CCNB2 (Figure 5D) IP. Interestingly, we observed abundant PAFAH1B1, CDK2 and CDK1 protein enrichment in the anti-CCNE1 IP (Figure 5E), while CDK2 and CDK1 protein were not enriched in the anti-CCNE2 IP (Figure 5F). These findings suggested that PAFAH1B1 might bind to CCNE1-CDK2-CDK1 protein complex to arrest the cell cycle at the G2/M phase.

### 3.6 *PAFAH1B1* disrupted the cell cycle in follicular development

**FIGURE 6:**
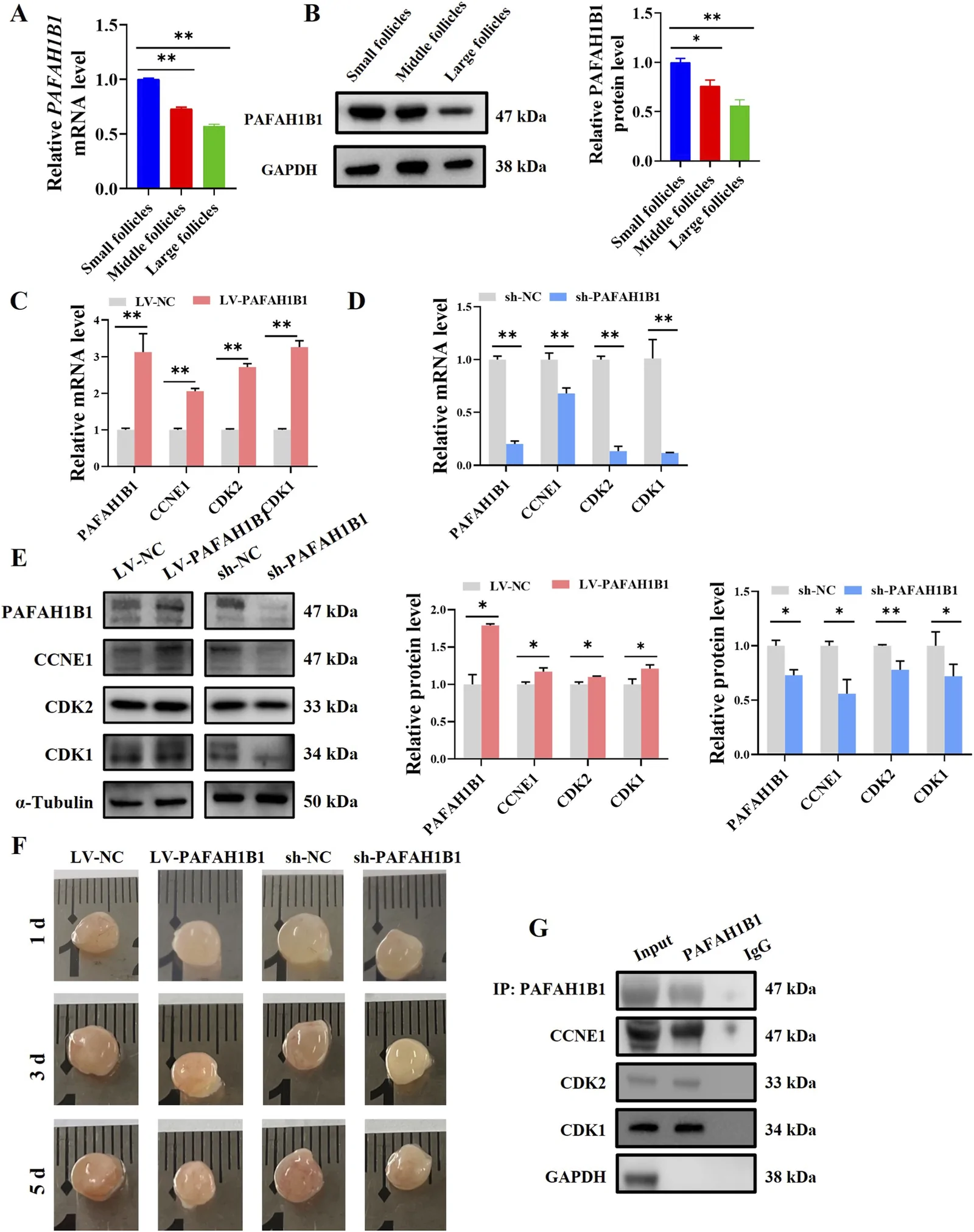
*PAFAH1B1* inhibited the follicular development in pigs. **(A)** Relative mRNA levels of *PAFAH1B1* in small (<3 mm), middle (3–5 mm), and large (>7 mm) follicles. **(B)** Protein levels of PAFAH1B1 in small, middle, and large follicles. **(C)** Relative mRNA levels of *PAFAH1B1*, *CCNE1*, *CCND1*, and *CDK1* in follicles transfected with LV-*PAFAH1B1*. **(D)** Relative mRNA levels of *PAFAH1B1*, *CCNE1*, *CCND1*, and *CDK1* in follicles transfected with sh-*PAFAH1B1*. **(E)** Protein levels of PAFAH1B1, CCNE1, CDK2, and CDK1 in follicles transfected with LV-*PAFAH1B1* and sh-*PAFAH1B1*. **(F)** The appearance of porcine follicles transfected with LV-*PAFAH1B1* or sh-*PAFAH1B1* on the 1st, 3rd, and 5th day. **(G)** The interaction between PAFAH1B1 and CCNE1, CDK2, or CDK1 protein in porcine follicles detected by CoIP. Data are shown as mean ± SEM from three independent biological replicates. *, *p* < 0.05. **, *p* < 0.01.

To investigate the effect of *PAFAH1B1* on porcine follicle development, we first analyzed its endogenous expression pattern. *PAFAH1B1* expression was significantly lower in large (>7 mm) and middle follicles (3–5 mm) than in small follicles (<3 mm) in mRNA (Figure 6A) and protein (Figure 6B) levels. This negative correlation with follicular size suggested a potential suppressive role for *PAFAH1B1* in follicular development. To functionally test this hypothesis, we employed lentiviral vectors to overexpress (LV-*PAFAH1B1*) or knockdown (sh-*PAFAH1B1*) *PAFAH1B1* in follicles. LV-*PAFAH1B1* significantly increased *PAFAH1B1* expression, while sh-*PAFAH1B1* showed the opposite results (Figures 6C–E). Additionally, LV-*PAFAH1B1* significantly increased *CCNE1*, *CCND1*, and *CDK1* mRNA levels (Figure 6C), and sh-*PAFAH1B1* showed the opposite results (Figure 6D). LV-*PAFAH1B1* significantly increased the protein levels of CCNE1 and CDK2, and sh-*PAFAH1B1* significantly decreased CCNE1, CDK2 and CDK1 protein (Figure 6E). Porcine follicles were treated with LV-*PAFAH1B1*, LV-NC, sh-*PAFAH1B1*, and sh-NC (Figure 6F). CoIP detection also showed that PAFAH1B1 bound to CCNE1, CDK2, and CDK1 proteins in porcine follicles (Figure 6G). These findings suggested that *PAFAH1B1* might inhibit follicular development by binding to *CCNE1*, *CDK2*, and *CDK1* protein in porcine follicles.

### 3.7 *Pafah1b1* inhibited follicular development in mice

**FIGURE 7:**
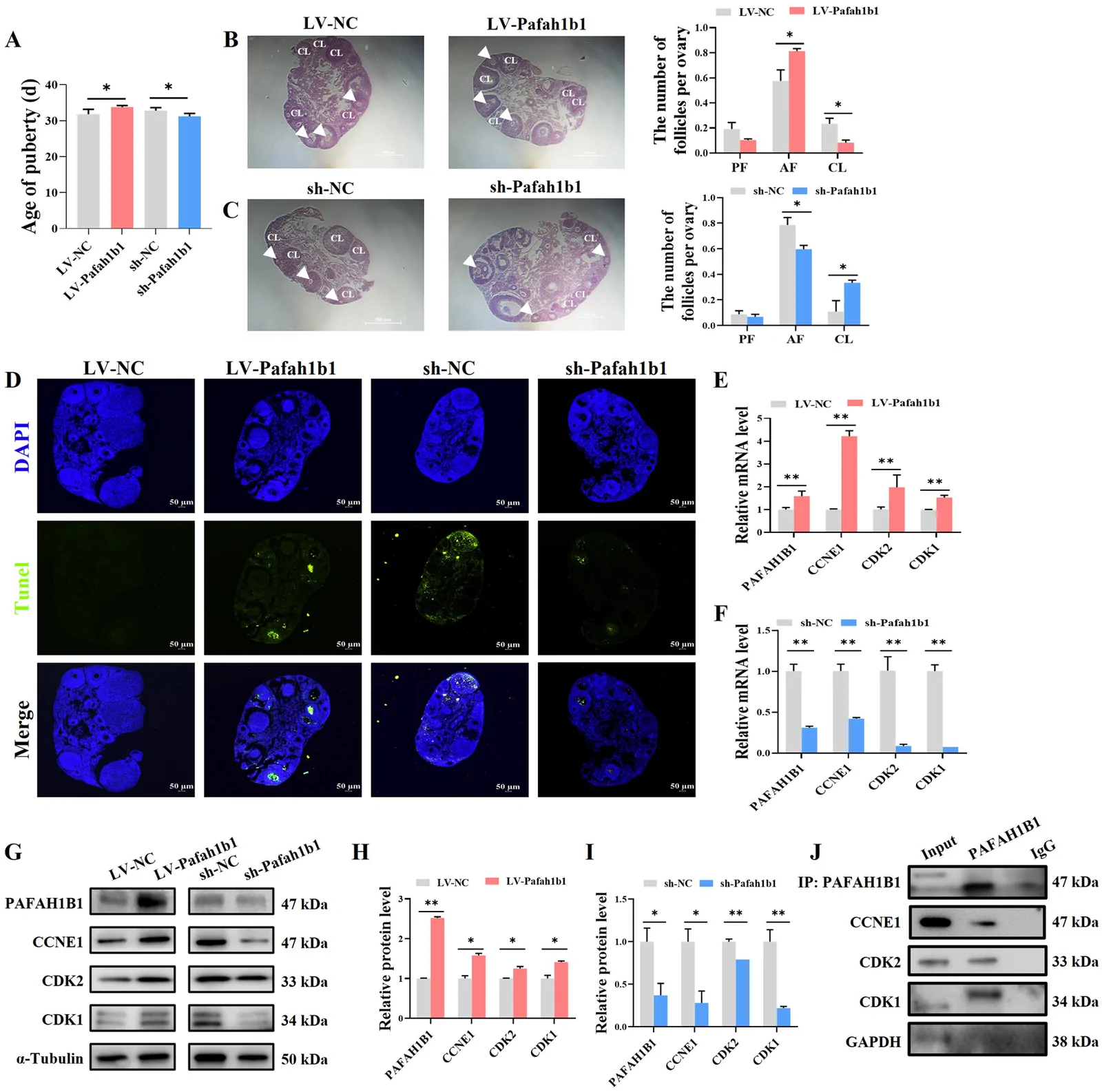
*Pafah1b1* inhibited follicular development in mice. **(A)** The age of puberty in mice following ovarian transfection with LV-*Pafah1b1* and sh-*Pafah1b1*. **(B)** The number of preantral follicles (PF), antral follicles (AF), and corpus luteum (CL) per ovary in mice transfected with LV-*Pafah1b1*. Scale bar, 500 μm. **(C)** The number of PF, AF, and CL per ovary in mice transfected with sh-*Pafah1b1*. Scale bar, 500 μm. **(D)** The apoptosis of follicular GCs in mice transfected with LV-*Pafah1b1* and sh-*Pafah1b1*. Scale bar, 50 μm. **(E)** Relative mRNA levels of *Pafah1b1*, *Ccne1*, *Cdk2*, and *Cdk1* in mouse ovary transfected with LV-*Pafah1b1*. **(F)** Relative mRNA levels of *Pafah1b1*, *Ccne1*, *Cdk2*, and *Cdk1* in mouse ovary transfected with sh-*Pafah1b1*. **(G)** Protein levels of PAFAH1B1, CCNE1, CDK2 and CDK1 in mouse ovary transfected with LV-*Pafah1b1* and sh-*Pafah1b1*. **(H)** Relative protein levels of PAFAH1B1, CCNE1, CDK2 and CDK1 in mouse ovary transfected with LV-*Pafah1b1.* **(I)** Relative protein levels of PAFAH1B1, CCNE1, CDK2 and CDK1 in mouse ovary transfected with sh-*Pafah1b1*. **(J)** The interaction of PAFAH1B1 and CCNE1, CDK2, or CDK1 protein in mouse ovary was detected by CoIP. Data are shown as mean ± SEM from three independent biological replicates. *p* values are stated in the figures. *, *p* < 0.05. **, *p* < 0.01.

To further investigate the role of *Pafah1b1* in regulating follicular development *in vivo*, we intraperitoneally injected 3-week-old C57BL/6J mice with *Pafah1b1* overexpression lentivirus (LV-*Pafah1b1*) and *Pafah1b1* knockdown lentivirus (sh-*Pafah1b1*). Then the mice were slaughtered at 7-weeks-old, compared to the control (31.8 ±1.4 d ), the mice injected with LV-*Pafah1b1* (33.8 ± 0.4 d) experienced a delay in vaginal opening and pubertal initiation significantly. Mice injected with sh- *Pafah1b1* (31.2 ±0.8 d ) exhibited a significantly earlier onset of puberty compared to the control group (32.8 ±0.8 d , Figure 7A). LV-*Pafah1b1*-injected mice showed a decrease in the number of corpora lutea (CL) and a marked increase in antral follicles (AF) (Figure 7B). Conversely, sh-Pafah1b1-injected mice exhibited an increase in CL and a marked decrease in AF (Figure 7C). LV-*Pafah1b1* promoted the apoptosis, and sh-*Pafah1b1* inhibited the apoptosis of GCs in mouse ovaries (Figure 7D). LV-*Pafah1b1* significantly increased the mRNA (Figure 7E) and protein (Figures 7G,H) levels of *Pafah1b1*, *Ccne1*, *Cdk2*, and *Cdk1*, and sh- *Pafah1b1* showed the opposite results (Figures 7F,G,I). CoIP detection also showed that PAFAH1B1 bound to CCNE1, CDK2, and CDK1 proteins in mouse ovaries (Figure 7J). These findings suggest that PAFAH1B1 may inhibit follicular development in mice by binding to CCNE1, CDK2, and CDK1 proteins.

### 3.8 *DNA* methylation modulated the transcription of *PAFAH1B1*

**FIGURE 8:**
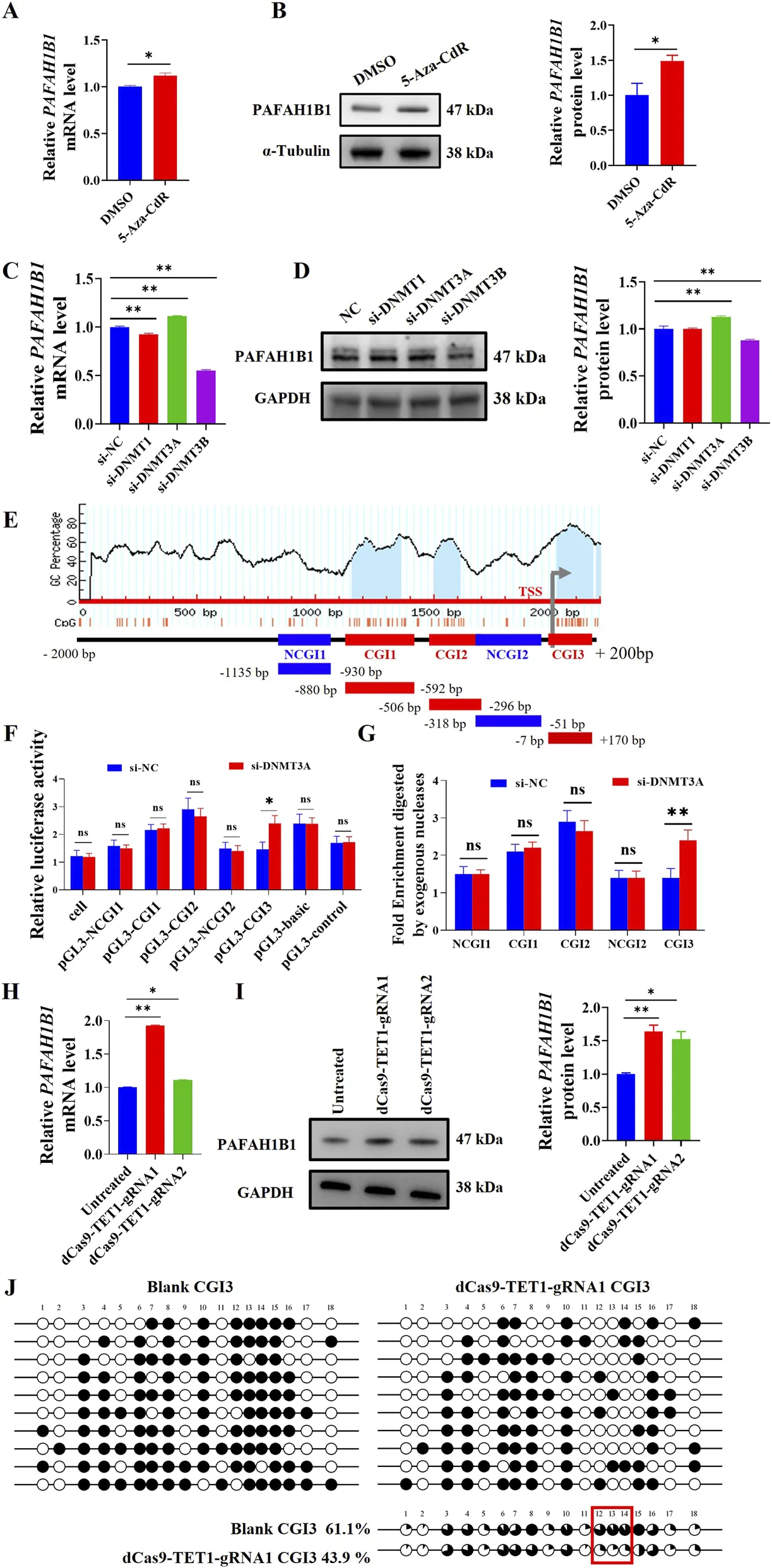
DNA methylation regulated the transcription of *PAFAH1B1*. **(A)** Relative mRNA levels of *PAFAH1B1* in GCs were treated with 5-Aza-CdR. **(B)** Protein levels of PAFAH1B1 in GCs were treated with 5-Aza-CdR. **(C)** Relative mRNA levels of *PAFAH1B1* in GCs transfected with si-*DNMT1*, si-*DNMT3A* and si-*DNMT3B*. **(D)** Protein levels of PAFAH1B1 in GCs transfected with si-*DNMT1*, si-*DNMT3A* and si-*DNMT3B*. **(E)** Schematic distribution of the CGIs and bisulfite sequencing primers in the promoter of *PAFAH1B1*. Blue region is the CGI. **(F)** Relative luciferase activity of *PAFAH1B1* promoter in GCs treated with si-*DNMT3A*. **(G)** Chromatin accessibility of *PAFAH1B1* promoter in GCs treated with si-*DNMT3A*. **(H)** Relative mRNA levels of *PAFAH1B1* in GCs treated with dCas9-TET1-gRNAs. **(I)** Protein levels of PAFAH1B1 in GCs treated with dCas9-TET1-gRNAs. **(J)**. Methylation status of CGI3 in GCs treated with dCas9-TET1-gRNAs. Data are shown as mean ± SEM from three independent biological replicates. *p* values are stated in the figures. *, *p* < 0.05. **, *p* < 0.01.

Our previous study showed that 5-Aza-CdR promotes GC apoptosis and inhibits follicular development. We also identified three CpG islands in the *PAFAH1B1* promoter, suggesting DNA methylation regulated its transcription. To test this, GCs were treated with 5-Aza-CdR, which significantly elevated *PAFAH1B1* mRNA and protein levels (Figures 8A,B). To identify the specific DNA methyltransferases that regulate the expression of *PAFAH1B1*, we interfered with the expressions of *DNMT1*, *DNMT3A*, and *DNMT3B*, and only si-*DNMT3A* remarkably elevated the mRNA (Figure 8C) and protein (Figure 8D) levels of *PAFAH1B1*. The promoter of *PAFAH1B1* contained three CpG islands (CGI) located at −880 bp to −592 bp, −506 bp to −296 bp, and −7 bp to +170 bp. To elucidate how DNA methylation regulates *PAFAH1B1* transcription, we segmented −2000 to +200 bp region on *PAFAH1B1* promoter into five different deletion fragments (Figure 8E). Deletion fragments included CGI1: −880 bp to −592 bp, CGI2: −506 bp to −296 bp, CGI3: −7 bp to +170 bp, NCGI1: −1135 bp to −930 bp, and NCGI2: −318 bp to −51 bp, were then cloned into pGL3 vector. We found si-*DNMT3A* significantly promoted the dual-luciferase activity of pGL3-CGI3 but showed an insignificant effect on pGL3-CGI1, pGL3-CGI2, pGL3-NCGI1, and pGL3-NCGI2 (Figure 8F). We validated a significant improvement in chromatin accessibility of CGI3 in GCs transfected with si-*DNMT3A* (Figure 8G). Furthermore, we utilized guide RNAs (gRNA) in conjunction with dCas9 fusion protein containing the catalytic domain of TET1 (dCas9-TET1) to promote the DNA hypomethylation of CGI3 (−7 bp to +170 bp). The targeting of dCas9-TET1 with either gRNA1 or gRNA2 resulted in a significant increase in the mRNA and protein levels of *PAFAH1B1* (Figures 8H,I), while dCas9-TET1-gRNA1 effectively suppressed DNA methylation levels within the CGI3 region (Figure 8J). Collectively, DNA hypomethylation contributed to increased chromatin accessibility within the CGI3 region of the *PAFAH1B1* promoter (−7 bp to +170 bp), resulting in activation of *PAFAH1B1* expression.

## 4 Discussion

The proliferation and apoptosis of GCs, as key processes in ovarian physiology, are involved in follicle growth (Hsueh et al., 2015). Abnormal and arrested cell cycle results in cellular senescence (Kumari and Jat, 2021). We demonstrated that *PAFAH1B1* bound to CCNE1-CDK2-CDK1 protein complex to arrest the cell cycle at the G2/M phase, thereby inhibiting the cell proliferation in our study. Furthermore, we identified that the transcription of *PAFAH1B1* was regulated by DNA methylation within the CGI3 region of its promoter, and that the demethylation of this region effectively reactivated *PAFAH1B1* expression. Aberrant GC proliferation directly affects the physiological characteristics of developing follicles and causes ovarian diseases such as ovarian cancer, infertility, and polycystic ovary syndrome (Wu et al., 2001). Excessive apoptosis of GCs leads to follicular atresia and blocks follicular development (Quirk et al., 2004).

In the scRNA-seq analysis of healthy and atretic porcine follicles, mural GCs showed the highest abundance among all cell types (Figures 1A,B). Studies showed that mural GCs are essential for follicle development and steroid hormone production. During the follicular phase, mural GCs synthesize and secrete estradiol (Dompe et al., 2021), which is essential for follicular growth, maturation, and ovulation (Fauser, 1997; Hu et al., 2023). Excessive apoptosis of mural GCs leads to follicular atresia (Fan et al., 2019; Fu et al., 2024). Moreover, we also found that the DEGs of mural GCs between healthy and atretic follicles were significantly enriched in pathways related to cell cycle, apoptosis, and ovarian steroidogenesis (Figure 1E). *PAFAH1B1* expression was significantly downregulated in mural GCs from atretic follicles compared with healthy follicles, while no significant changes were observed in theca cells or cumulus GCs (Figure 1F). *PAFAH1B1*, a gene involved in cell cycle progression in other cell types, has remained largely unexplored in GC function and follicular development. We therefore hypothesized that *PAFAH1B1* may regulate GC cycle progression. Consistent with this hypothesis, we found that *PAFAH1B1* inhibited proliferation (Figure 3A) and promoted the apoptosis of GCs (Figure 3I). Our findings indicate that G2/M phase arrest is associated with suppressed proliferation and enhanced apoptosis in GCs. Other studies also showed that A35 induces DNA damage and G2/M phase arrest, leading to HepG2 cell apoptosis (Zhao et al., 2018). Similarly, knockdown of HDAC9 inhibits G2/M phase arrest and thereby suppresses apoptosis in tubular epithelial cells (Zhang et al., 2023), while Naringenin induces cell cycle arrest, apoptosis and autophagy through enhanced ROS production (Chang et al., 2024). Furthermore, *PAFAH1B1* significantly decreased the distribution of GCs in G1 phase and increased cell cycle arrest at the G2/M phase. *PAFAH1B1* markedly upregulated pro-G1/S transition gene (*CDK2*, *CDK4*, *CCND1*, *CCNE1*, and *CCNE2*) and pro-G2/M phase gene (*CDK1*), whereas it downregulated *CCNB1* and *CCNB2* (Figure 4). Additionally, *CDCA8* knockdown in CAL-27 cells promotes apoptosis, inhibits proliferation and migration, and reduces the levels of *CDK1* and *CDK2* (Zhu et al., 2025). CDC25C knockdown in U87MG cells suppresses proliferation, induces G2/M arrest, and inhibits the *CCNB1* (Qiao et al., 2025). Collectively, these observations indicate that G2/M dysregulation is linked to cell apoptosis across various cell types. We also found that *PAFAH1B1* affected vascular degeneration in porcine ovaries (Figure 6F) and inhibited follicle development in mouse (Figure 7). We identified *PAFAH1B1* as a potential regulator of cell cycle progression in GCs and established a correlative link between *PAFAH1B1* expression and follicular atresia. Future rescue experiments are needed to clarify the role of *PAFAH1B1*-mediated G2/M arrest in follicular degeneration.

We further investigate how *PAFAH1B1* regulates GC cell cycle progression. CoIP showed that abundant PAFAH1B1. CDK2 and CDK1 enriched in anti-CCNE1 IP (Figure 5E). The binding between PAFAH1B1 and CCNE1, CDK2, and CDK1 proteins was also observed in porcine follicles (Figure 6G) and mouse ovaries (Figure 7J). To assess the evolutionary conservation of this regulatory mechanism, we examined PAFAH1B1 sequence conservation across species. Porcine PAFAH1B1 protein shares 84.2% and 90.5% amino acid identity with its human and mouse orthologs, respectively. The core WD40-repeat domains exceed 96% conserved across all three species, indicating strong structural conservation. Collectively, these findings indicate that *PAFAH1B1*-mediated regulation of cell cycle-related proteins is evolutionarily conserved. The cell cycle is divided into G1, S, G2, and M phases. The transition of cell cycle is controlled by various CDKs and their activated cyclin subunits (Bertoli et al., 2013). *CCNE1*, the first identified member of the E-type cyclin family, has been verified to promote S-phase entry and progression by binding to the *CDK2* kinase to form an activated complex (Ohtsubo and Roberts, 1993; Resnitzky et al., 1994). *CCNE1* can bind to *CDK1* and activate it *in vivo* (Aleem et al., 2005). The role of cyclin-dependent kinase complexes CDK2 and CDK1, in the cell division cycle is crucia (Golsteyn, 2005). *CDK2* mutations inhibit the cellular transition to S phase (Hochegger et al., 2008). *CDK1* affects cell proliferation and premature senescence (Diril et al., 2012). The S and M phases of the cell cycle are driven by CDK2 (Tadesse et al., 2019). Therefore, we speculated that *PAFAH1B1* might bind to CCNE1-CDK2-CDK1 protein complex to arrest the cell cycle at the G2/M phase and decrease in G1 phase cells. Our results establish *PAFAH1B1* as an interacting partner of this complex and suggest its potential utility as a biomarker for future studies on follicular development.

DNA methylation, typically associated with gene silencing, regulates gene transcription by inhibiting the binding of transcription factors to gene regulatory regions (Moore et al., 2013; Zhu et al., 2016). 5-Aza-CdR, forms stable covalent complexes with DNMTs, thereby downregulating their activity and blocking DNA methylation, leading to gene activation and influencing cellular processes (Zhang et al., 2016; Zhu et al., 2004). For instance, in gastric cancer cell lines, 5-Aza-CdR decreases DNA methylation at the *RSPO2* promoter and promotes its expression (Wilhelm et al., 2017). In our study, we found that DNA hypomethylation in CGI3 region (−7 bp to +170 bp) significantly promoted the chromatin accessibility and transcription of *PAFAH1B1* (Figures 8F,G). Consistently, our previous RNA-seq analysis showed that 5-Aza-CdR treatment significantly upregulated *PAFAH1B1* transcription in GCs compared with DMSO controls (Zhou et al., 2023). Moreover, a predicted SP1-binding motif within the CGI3 region was identified through JASPAR and TRANSFAC. SP1 is a well-characterized transcriptional activator that preferentially binds to GC-rich motifs (Yang et al., 2021) and governs housekeeping (Bhargavan et al., 2024; Pan et al., 2024) and tissue-specific genes (Gao et al., 2024; Zhang et al., 2024) by interacting with unmethylated CGI. We thus hypothesized that CGI3 hypomethylation facilitates SP1 recruitment to the *PAFAH1B1* promoter, driving its transcription. Future ChIP-qPCR can be utilized to validate SP1 occupancy at CGI3 and its downstream effects on *PAFAH1B1* transcription, GC apoptosis, cell cycle progression, and follicular development.

Several limitations of this study should be acknowledged. Although our CoIP data demonstrated a physical interaction between PAFAH1B1 and the cell cycle regulators CCNE1, CDK2, and CDK1, whether this association is direct or bridged by other factors remains to be determined. The precise stoichiometry and architecture of the complex await further elucidation. Another limitation is that while our findings indicated that CGI3 demethylation promoted *PAFAH1B1* transcription and that *PAFAH1B1* inhibited GC proliferation and promoted apoptosis, these two lines of evidence were not directly linked. Thus, rescue experiments combining dCas9-TET1-mediated demethylation with *PAFAH1B1* knockdown are required to firmly establish the causal relationship between CGI3 demethylation and *PAFAH1B1*-mediated GC functional outcomes. A further limitation is that all molecular mechanism experiments, including CoIP, promoter activity assays, chromatin accessibility, and dCas9-TET1-mediated demethylation analyses, were performed exclusively in the COV434 human granulosa tumor cell line. As a transformed cell line, COV434 may not fully recapitulate the cell cycle and DNA methylation dynamics of normal primary granulosa cells. Therefore, future studies employing primary porcine or murine GCs will be valuable to substantiate this regulatory axis and to better define its physiological relevance in ovarian development. Despite these limitations, our findings provide mechanistic insights into the role of *PAFAH1B1* in GC biology and lay a foundation for future investigations into the epigenetic regulation of follicular development.

## 5 Conclusion

**FIGURE 9:**
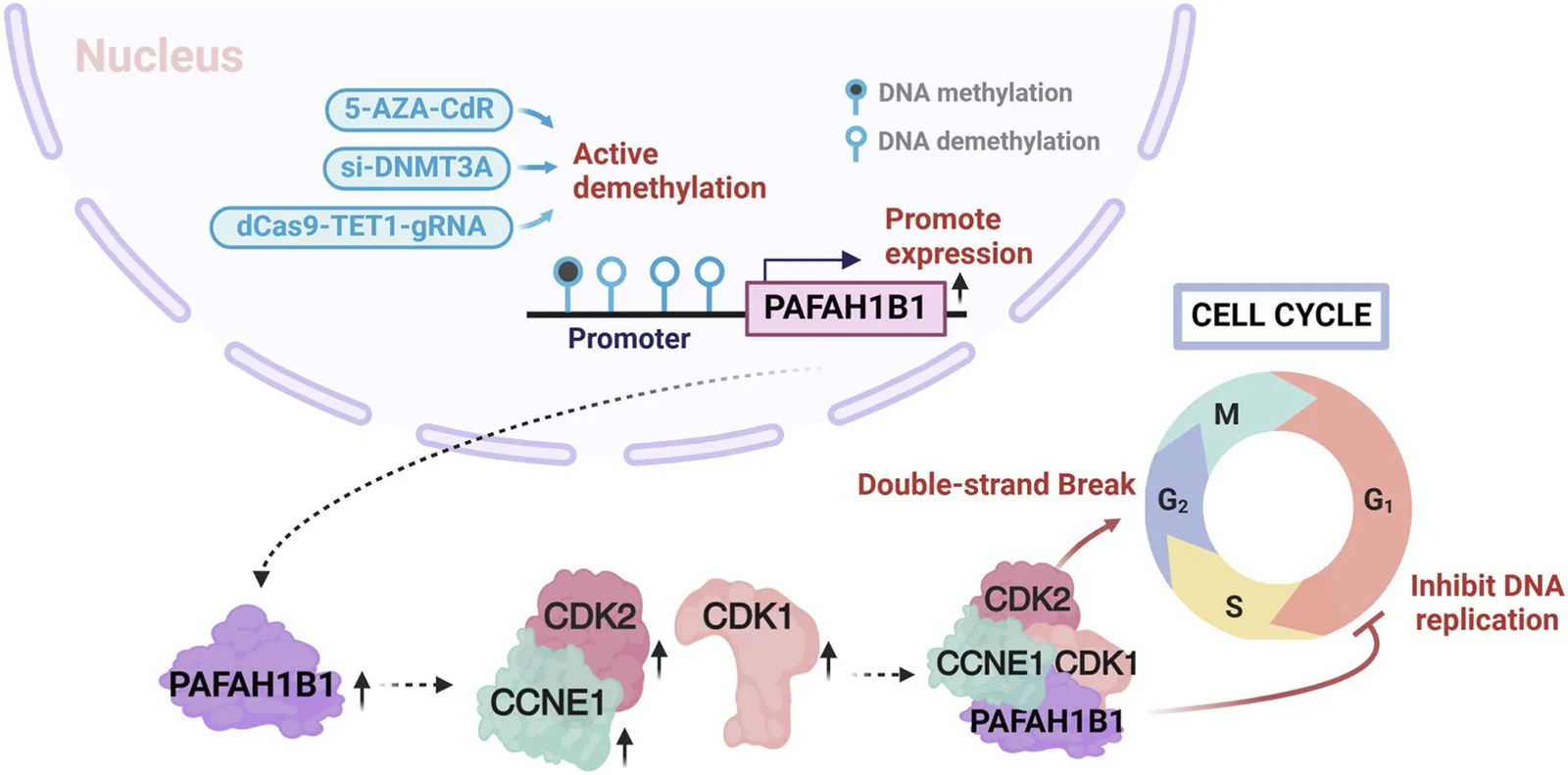
Schematic diagram of DNA methylation mediating *PAFAH1B1* transcription to regulate the cell cycle of GCs.

In conclusion, we found that the demethylation of CGI (−7 bp to +170 bp) within the *PAFAH1B1* promoter significantly promoted the transcription of *PAFAH1B1*. Additionally, *PAFAH1B1* formed a protein complex with CCNE1-CDK2-CDK1, which facilitated cell cycle arrest in the G2 phase. This, in turn, inhibited cell proliferation and promoted apoptosis in GCs (Figure 9).

## Data Availability

The original contributions presented in the study are included in the article/supplementary material, further inquiries can be directed to the corresponding authors.
